# In silico design of novel precision vaccine targeting sclerostin epitopes for osteoporosis prevention and treatment

**DOI:** 10.3389/fimmu.2025.1644437

**Published:** 2025-12-01

**Authors:** Jianzhou Luo, Tailin Wu, Bin Guan, Lin Li, Zili Yang, Huiren Tao

**Affiliations:** 1 Department of Orthopedics, Shenzhen University General Hospital, Shenzhen University Health Science Center, Shenzhen University, Shenzhen, Guangdong, China; 2 The Key Laboratory of Biomedical Information Engineering of Ministry of Education, School of Life Science and Technology, Xi’an Jiaotong University, Xi’an, Shaanxi, China; 3 Orthopedic Centre, the University of Hong Kong Shenzhen Hospital, Shenzhen, Guangdong, China; 4 Preventive Health Care Section, the Health Service Center of Weifang Community, Shanghai, China

**Keywords:** osteoporosis, sclerostin (SOST), romosozumab (ROMO), vaccine, translocation domain of diphtheria toxin (DTT)

## Abstract

**Background:**

Osteoporosis has become an increasingly pressing global public health challenge. Monoclonal antibody romosozumab (ROMO), which targets sclerostin (SOST), a critical inhibitor of bone formation, demonstrates considerable therapeutic efficacy. However, its relatively high cost and potential cardiovascular risks may hinder broader clinical application. Current preventive measures remain inadequate.

**Methods:**

This study presents a novel, cost-effective osteoporosis vaccine with dual preventive and therapeutic capabilities, derived from the high-affinity binding epitope of ROMO to SOST. ELISA screening determined that the SOST_131–163_ region within loop3 domain serves as the primary epitope for ROMO, suggesting a role in skeletal regulation with minimal impact on cardiovascular system. SOST_131–163_ was conjugated to the diphtheria toxin translocation domain (DTT) to create novel SOST-targeted vaccines.

**Results:**

Immunogenicity assays demonstrated that both DDT-SOST_(131-163)3_ (DS_3_) and DDT-SOST_(131-163)5_ (DS_5_) elicited strong IgG2 antibody responses comparable to ROMO. Molecular docking studies indicated strong affinities of DS_3_ and DS_5_ for Toll-like receptor 2 (TLR2), enhancing TLR2-mediated humoral B-cell immunity and eliciting synergistic T-helper cell responses. Recombinant expression in Escherichia coli confirmed the successful production of DS_3_ and DS_5_, with molecular weights of 31.8 kDa and 40.3 kDa, respectively. *In vivo* experiments showed that the vaccines effectively induced high-titer anti-SOST antibodies in mice, overcoming immune tolerance. Additionally, cell-based assays indicated that antiserum from vaccinated mice inhibited osteoclast differentiation and promoted osteoblast mineralization.

**Conclusion:**

The SOST-targeted vaccination strategy offers a promising and cost-effective approach for the early prevention and sustained management of osteoporosis, demonstrating substantial potential for clinical translation.

## Introduction

Osteoporosis (OP) is a prevalent degenerative bone disease defined by diminished bone mass and an elevated risk of fractures, posing a significant global health challenge (1, 2). It affects approximately one-third of women and one-fifth of men over the age of 50, with prevalence anticipated to rise population ages (2, 3). The disorder results from an imbalance in bone remodeling, where bone resorption outpaces bone formation. Current therapies primarily focus on promoting bone formation, such as teriparatide, or inhibiting resorption with agents like alendronate and denosumab (4). Although romosozumab (ROMO), a dual-action monoclonal antibody, has demonstrated promising efficacy, its high cost and cardiovascular adverse events may limit broader application (1, 5, 6). Furthermore, existing preventative strategies do not effectively address early intervention, underscoring the pressing need for innovative therapies (1).

Sclerostin (SOST) is a critical negative regulator of osteoblast differentiation, primarily inhibiting the Wnt signaling pathway, thus decreasing bone formation and indirectly promoting osteoclastogenesis (7). An agent targeting SOST, such as ROMO, offers a dual mechanism for modulating bone dynamics (8); however, its antibody-based design poses challenges in both safety and affordability (5, 9). Consequently, there is growing interest in vaccine-based strategies aimed at achieving safe, sustained preventive and therapeutic effects through the induction of long-lasting endogenous antibody production (10, 11). Nonetheless, the development of SOST vaccines encounters two primary hurdles: identifying effective antigenic epitopes and overcoming immune tolerance to self-proteins (12).

Building upon our previous successes in addressing immune tolerance in osteoporosis vaccine development (10), we propose a novel vaccine strategy that integrates the SOST protein with a diphtheria toxin translocation domain (DTT) as an adjuvant scaffold. Our approach commenced with the identification of the high-affinity binding domain of SOST through ROMO, followed by its conjugation to DTT protein to create a subunit vaccine. We performed comprehensive physicochemical characterization, validated mass producibility via recombinant expression in E. coli, and evaluated immunogenicity along with *in vivo* efficacy in antibody induction. This strategy is designed to achieve three primary objectives: (1) Confirming antigen validity by identifying effective SOST epitopes for ROMO targeting; (2) Overcoming immune tolerance with DTT scaffold to enhance antibody induction; and (3) Developing a cost-effective and scalable osteoporosis vaccine to enable early intervention and sustained therapeutic benefits.

## 2 Materials and methods

### 2.1 SOST peptide fragments

Peptides were synthesized based on the human SOST (GenBank: AAK16158.1) fragment encompassing amino acids 24 to 211, excluding the signal peptide, yielding approximately 30-amino-acid segments. An indirect enzyme-linked immunosorbent assay (ELISA) was performed to identify a high-affinity SOST peptide fragment for ROMO. The polypeptides were synthesized by Sangon Biotech (Shanghai, China).

### 2.2 Enzyme-linked immunosorbent assay (ELISA) for peptide screening

96-well plates (Abcam, Cambridge, MA, USA) were coated with 100 µL of 1 µg/mL human SOST-synthesized polypeptides in the provided coating buffer overnight at 4 °C. Following coating, the plates were washed with 1× washing buffer and subsequently blocked using 1× blocking buffer. Diluted ROMO (AntibodySystem, Schiltigheim, France) was then added to the wells and incubated for 2 hour at 37 °C. After additional washing, 100 µL of 1:10,000 diluted horseradish peroxidase (HRP)-labeled goat anti-human antibodies (Bioss, Beijing, China) was introduced to the wells, followed by another 2-hour incubation at 37 °C. Finally, 100 µL of TMB was dispensed into each well, and after a 20-minute incubation at 37 °C, the absorbance was measured at 450 nm.

The high-affinity SOST peptide fragment for ROMO was then identified using ELISA. The binding affinity of the high-affinity SOST fragment to the heavy and light chains of ROMO was assessed using the HawkDock server (13), while interactions between the SOST fragment and ROMO were analyzed with PDBsum (14).

### 2.3 Prediction of T cell and B cell epitope

Prediction of major histocompatibility complex (MHC) class I-restricted cytotoxic T lymphocyte (CTL) epitopes for the high-affinity SOST fragment was performed using NetMHCpan 4.1 EL tool (15). A comprehensive analysis of 9-mer epitopes was conducted across the A1, A2, A3, A24, and B7 supertypes. Epitopes with a percentage rank (< 0.5%) were categorized as strong binders (SB), whereas those with a percentage rank (< 2%) were classified as weak binders (WB). In parallel, the identification of helper T lymphocyte (HTL) epitopes, comprising 15-mer peptides that bind to MHC class II, was accomplished using the NetMHCIIpan 4.1 EL server (16), with strong binders defined as having a percentage rank (< 1%) and weak binders defined as having a percentage rank (< 5%).

To predict linear B cell epitopes within the high-affinity SOST fragment, we employed BepiPred2.0 tool (17), applying a default filtering threshold of 0.5. Additionally, conformational B cell epitopes for both the screening peptide and the designed vaccines were predicted using the ElliPro application (18), with a minimum score threshold set at 0.5 and a maximum distance of 6 Angstroms.

### 2.4 Construction and prediction of candidate vaccines

The high-affinity SOST fragment was selected as the target antigen for the development of a recombinant subunit vaccine. Based on our previous experimental experience (10), the DTT fragment (amino acids 203–378; WP_371890660.1) was chosen as immune scaffold to facilitate conjugation with SOST fragment, thereby enhancing immune recognition and promoting antibody production. The DTT scaffold was conjugated with varying copy numbers of SOST peptide, ranging from 0 to 5, to generate a series of chimeric molecules. These conjugations were connected via a (GGGGS)_2_ flexible linker to ensure optimal conformational flexibility. The resulting vaccine candidates were designated as DDT-SOST_(131-163)0_ (DS_0_) to DDT-SOST_(131-163)5_ (DS_5_), with the numerical suffix denoting specific number of SOST peptide fragments fused to DTT scaffold.

Tertiary structure of candidate vaccines was predicted using AlphaFold2 server (19), based on their amino acid sequences. Five structural models were generated, demonstrating close alignment with experimental accuracy. Top-ranked model was selected for further analysis. The quality of vaccine structure was assessed using the predicted Local Distance Difference Test (pLDDT).

### 2.5 Immune response simulation

To evaluate the immunogenic potential of candidate vaccines, we employed the C-ImmSim server (20), a platform capable of simulating immune responses. This computational tool mimics the activation of B and T lymphocytes following hypothetical vaccine administration, allowing for the exploration of immune response dynamics. The simulation parameters were configured as follows: Random Seed = 12,345, Simulation Volume = 10, Simulation Steps = 240, and HLA selections: A0101, B0702, and DRB1_0101. The simulation framework was designed to include three administrations of 400 antigens, each spaced by a two-week interval. Each time step was delineated to represent an elapsed duration of 8 hours in real-world time, leading to time periods set at 1, 42, and 84. The simulation predicted the cellular immune responses provoked by candidate vaccines, encompassing antibody production, B cell and T cell activation, and cytokine release. The vaccine demonstrating the highest titer of IgG2 antibody were subsequently selected for further analysis, given that the IgG2 subtype is known to mediate the function against SOST in ROMO (21).

### 2.6 Prediction of immunological and physicochemical properties

Immunological properties of the selected vaccines were systematically evaluated. Allergenicity assessments were performed using AllerTOP v.2.1 server (22), while antigenic potential was analyzed with VaxiJen v2.0 server (23). To assess solubility of the vaccines, SOLpro server (24) was employed. Additionally, the physicochemical properties—including chemical formula, total atom count, molecular weight, theoretical isoelectric point (pI), half-life, instability index, aliphatic index, and the grand average of hydropathicity (GRAVY)—were predicted using ExPASy ProtParam tool (25).

### 2.7 Prediction and analysis of secondary structure

Secondary structure elements of the selected vaccines, including α-helices, extended strands, β-turns, and random coils, were predicted using SOPMA server (26) and PSIPRED web server (27). For these predictions, all parameters were maintained at their default settings. Additionally, the solubility characteristics of the selected vaccines were assessed using Protein-Sol server (28).

### 2.8 Refinement and validation of tertiary structure

Top-ranked model of tertiary structure for the selected vaccines, generated by AlphaFold2, was refined using GalaxyRefine web server (29). This refinement yielded reliable core structures based on multiple templates, while less reliable loops and terminal regions were constructed through optimization-based modeling. The structural quality of the refined vaccine model was further assessed using ProSA-web (30), ERRAT (31) and PROCHECK (32).

### 2.9 Molecular docking and molecular dynamic simulations

Molecular docking analyses were conducted using HawkDock server (13) to evaluate the interactions between vaccine candidates and Toll-Like Receptor 2 (TLR2) immune receptor (PDB ID: 6NIG). This platform organizes docking models based on surface complementarity and clustering characteristics. The highest-ranking model derived from the docking evaluations was selected for further analysis and visualized with PyMOL software. Binding energy and interaction surfaces within the docking complex were assessed using Prodigy (33), PDBePISA (34), and PDBsum (14).

Molecular dynamics simulations of the vaccine-TLR2 docking complex were performed utilizing the internal coordinate normal mode analysis server (iMODS) (35). This platform employs Normal Mode Analysis (NMA) in internal coordinates to identify collective motions that are critical for the functional dynamics of macromolecules. iMODS provides interactive tools for visualizing these modes, including vibration analysis, motion animations, and morphing trajectories.

### 2.10 Vaccines cloning, expression and immunization

Codon-optimized cDNA sequences for the selected vaccine candidates were generated in silico using the Java Codon Adaptation Tool (JCAT) (36). Optimized sequences were then cloned into pSmartI plasmids. Following cloning, the recombinant plasmids were transformed into Escherichia coli BL21 (DE3) for protein expression. The resulting recombinant proteins were purified through a two-step chromatography process, which involved ion exchange chromatography followed by gel filtration chromatography. Purity and quality of protein products were assessed using 10% sodium dodecyl sulfate-polyacrylamide gel electrophoresis (SDS-PAGE) (Sangon Biotech, Shanghai, China).

For immunization studies, C57BL/6J mice (purchased from Guangdong Medical Laboratory Animal Center, China) received two subcutaneous injections of 200 µg of each vaccine candidate, administered two weeks apart(n=3). Freund’s Complete Adjuvant was used for the initial dose, while Freund’s Incomplete Adjuvant (Sigma, USA) was employed for the booster injection. Blood samples were collected five weeks after the final immunization, and anti-SOST antibody titers were measured using ELISA.

### 2.11 Detection of anti-SOST antibodies in vaccinated mice

The antiserum from vaccine-immunized mice was obtained. The titers of specific anti-SOST antibodies were assessed using an indirect ELISA. In brief, 1 µg/mL of human SOST protein (MedChemExpress Inc.) was coated onto the wells of MaxiSorp microtiter plates (Thermo Fisher Scientific Inc.) and incubated overnight at 4°C. Mouse serum samples were diluted 1:200 in sample dilution buffer and added to the pre-coated plates, followed by incubation at room temperature for 2 hours. After washing the plates with washing buffer, bound IgG was detected using a horseradish peroxidase-conjugated goat anti-mouse IgG antibody (1:10,000, Abcam). The absorbance was measured at 450 nm using a Multiskan FC microplate reader (Thermo Fisher Scientific, San Jose, USA).

### 2.12 Assessment of T cell immune responses post-vaccine stimulation

Splenocytes were isolated immediately post-euthanasia via mechanical dissociation of the spleen tissue. Mononuclear cells were then separated using density gradient centrifugation with a murine spleen mononuclear cell isolation kit (Solarbio, Beijing, China). Isolated cells were resuspended at a concentration of 1×10^6 cells/mL in RPMI 1640 medium supplemented with 10% fetal bovine serum. Cells were subsequently stimulated *in vitro* with recombinant SOST protein (100 ng/mL), vaccine protein (100 ng/mL), or PBS as a control, and incubated for 48 hours at 37 °C in a humidified atmosphere containing 5% CO_2_. Post-incubation, culture supernatants were collected and analyzed for cytokine concentrations (IL-4, IL-10, and IFN-γ) using ELISA kits (Meimian Industrial Co., Ltd., China), following the manufacturer’s instructions.

### 2.13 *In vitro* validation of anti-SOST antiserum function

Functional activity of anti-SOST antiserum from vaccine-immunized mice was evaluated using primary osteoclasts and osteoblasts. Mice were euthanized via CO_2_ inhalation, starting with a flow rate of 10% of chamber volume per minute to gradually increase CO_2_ concentration to 30%, inducing unconsciousness. Once righting reflex was lost, the flow rate was increased to 30% per minute to maintain a CO_2_ concentration of ≥70% for 5 minutes, ensuring humane euthanasia. All procedures complied with animal welfare guidelines. Primary osteoclasts were isolated from tibiae and femora of 8-week-old C57BL/6J mice. Bone marrow mononuclear cells were extracted using an isolation kit, filtered, and cultured in α-MEM supplemented with 50 ng/mL M-CSF and 80 ng/mL sRANKL (PeproTech) for 4–6 days to induce differentiation. During the second medium change, anti-SOST antiserum and 100 ng/mL recombinant SOST protein (Novoprotein) were added. Osteoclast differentiation was confirmed by TRAP staining (Servicebio). Primary osteoblasts were obtained from bone marrow stromal cells, and the MC3T3-E1 subclone 14 osteoblast cell line (purchased from Pricella Biotechnology Co., Ltd.) was cultured in osteogenic medium. Co-cultures of osteoblasts with anti-SOST antiserum and 200 ng/mL SOST were established, and mineralization was evaluated on day 21 via Alizarin Red S staining (Solarbio). The culture medium was refreshed every 2–3 days throughout the experiment.

### 2.14 Statistical analysis

Data are presented as means ± standard deviation (SD). Differences between two independent groups were analyzed using one-way ANOVA followed by Tukey’s multiple comparisons test. Data visualization was conducted utilizing GraphPad Prism software version 10 (GraphPad Software, San Diego, CA, USA). A p-value of less than 0.05 was considered statistically significant.

## 3 Results

### 3.1 Screening of high-affinity SOST epitope for ROMO binding

**Figure 1:**
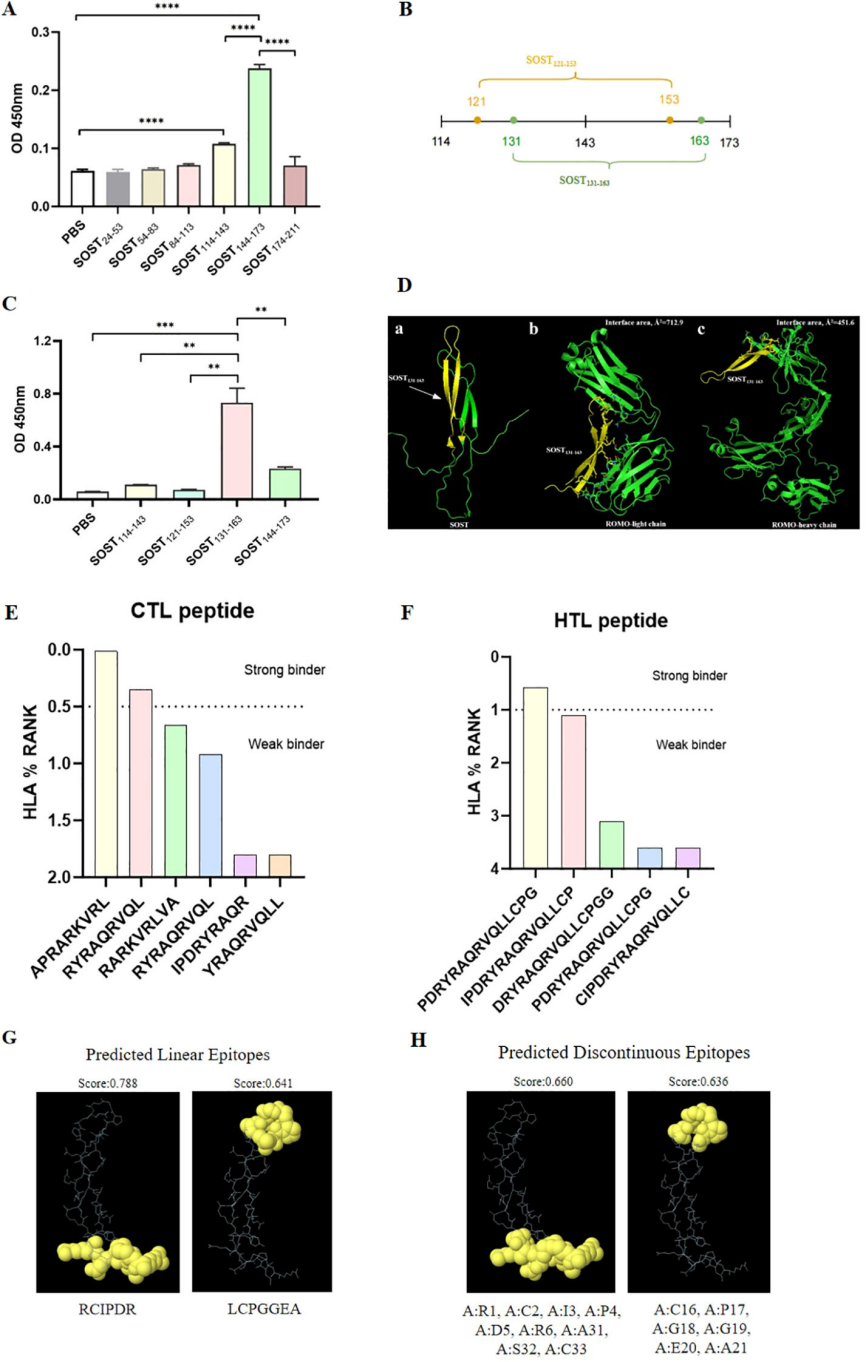
Screening and analysis of high-affinity epitopes on SOST. **(A)** ELISA experiments were conducted to identify SOST fragments with strong binding affinity for ROMO, revealing that SOST_114–143_ and SOST_143–173_ exhibit significantly higher affinity (*P*<0.01). **(B)** A schematic diagram delineating the binding functional regions associated with the high-affinity fragments of SOST. **(C)** ELISA results indicate that SOST_131–163_ displays the highest affinity for ROMO (*P*<0.01), thereby identifying it as a potent functional epitope of SOST. **(D-a)** SOST_131–163_ fragment (highlighted in yellow) is located within the loop3 domain of SOST protein. **(D-b)** Docking studies indicate that SOST_131–163_ fragment interacts with ROMO light chain, yielding a binding free energy of -25.8 kcal/mol and an interface area of 712.9 Å². **(D-c)** Additionally, SOST_131–163_ fragment can bind to the ROMO heavy chain, resulting in a binding free energy of -33.19 kcal/mol and an interface area of 451.6 Å². **(E)** CTL epitopes within SOST_131–163_ sequence include two strong binder epitopes and four weak binder epitopes. **(F)** HTL epitopes in SOST_131–163_ sequence comprise one strong binder epitope and four weak binder epitopes. Predictions of B cell epitopes for SOST_131–163_ sequence are illustrated, including predicted linear B cell epitopes **(G)** and predicted discontinuous B cell epitopes **(H)**.

To identify potential interaction sites of SOST with ROMO for development of recombinant subunit vaccines, we initially fragmented SOST protein into six peptides, each comprising approximately 30 amino acids. Screening through ELISA pinpointed two peptides, SOST_114–143_ and SOST_144-173_, that exhibited high-affinity binding to ROMO (**Figure 1A**). We further dissected the identified region (amino acids 114-173) into four peptides based on their biological properties (**Figure 1B**). A subsequent ELISA revealed that SOST_131–163_ peptide served as a specific epitope with substantial affinity for ROMO (**Figure 1C**), located within loop3 domain of SOST (**Figure 1D-a**).

Molecular docking studies indicated that SOST_131–163_ fragment interacts with the light chain of ROMO’s variable domain, establishing 4 hydrogen bonds and 105 non-bonded contacts, resulting in a binding free energy of -25.8 kcal/mol and an interface area of 712.9 Å² (**Figure 1D-b**, **Supplementary Figure S1A**). Furthermore, SOST_131–163_ demonstrated affinity for the heavy chain’s variable domain, forming 2 salt bridges and 90 non-bonded contacts, with a binding free energy of -33.19 kcal/mol and an interface area of 451.6 Å² (**Figure 1D-c**, **Supplementary Figure S1B**). These docking results corroborate that SOST_131–163_ is a critical and distinctive peptide for ROMO, aligning with our ELISA observations.

Identification of immunodominant epitopes is pivotal for effective vaccine design. In this study, NetMHCpan 4.1 EL tool was utilized to predict six cytotoxic T lymphocyte (CTL) epitopes in SOST_131–163_ fragment, consisting of four weak binders and two strong binders (**Figure 1E**). Additionally, NetMHCIIpan 4.1 EL server was employed to forecast five helper T lymphocyte (HTL) epitopes, comprising four weak binders and one strong binder (**Figure 1F**). For the prediction of B cell epitopes, linear epitopes were analyzed using BepiPred 2.0 tool, resulting in the identification of two distinct epitopes within SOST_131–163_ fragment (**Figure 1G**). Furthermore, conformational B cell epitopes were evaluated using ElliPro tool, which yielded two additional epitopes (**Figure 1H**).

### 3.2 Construction and immunogenicity prediction of candidate vaccines

**Figure 2:**
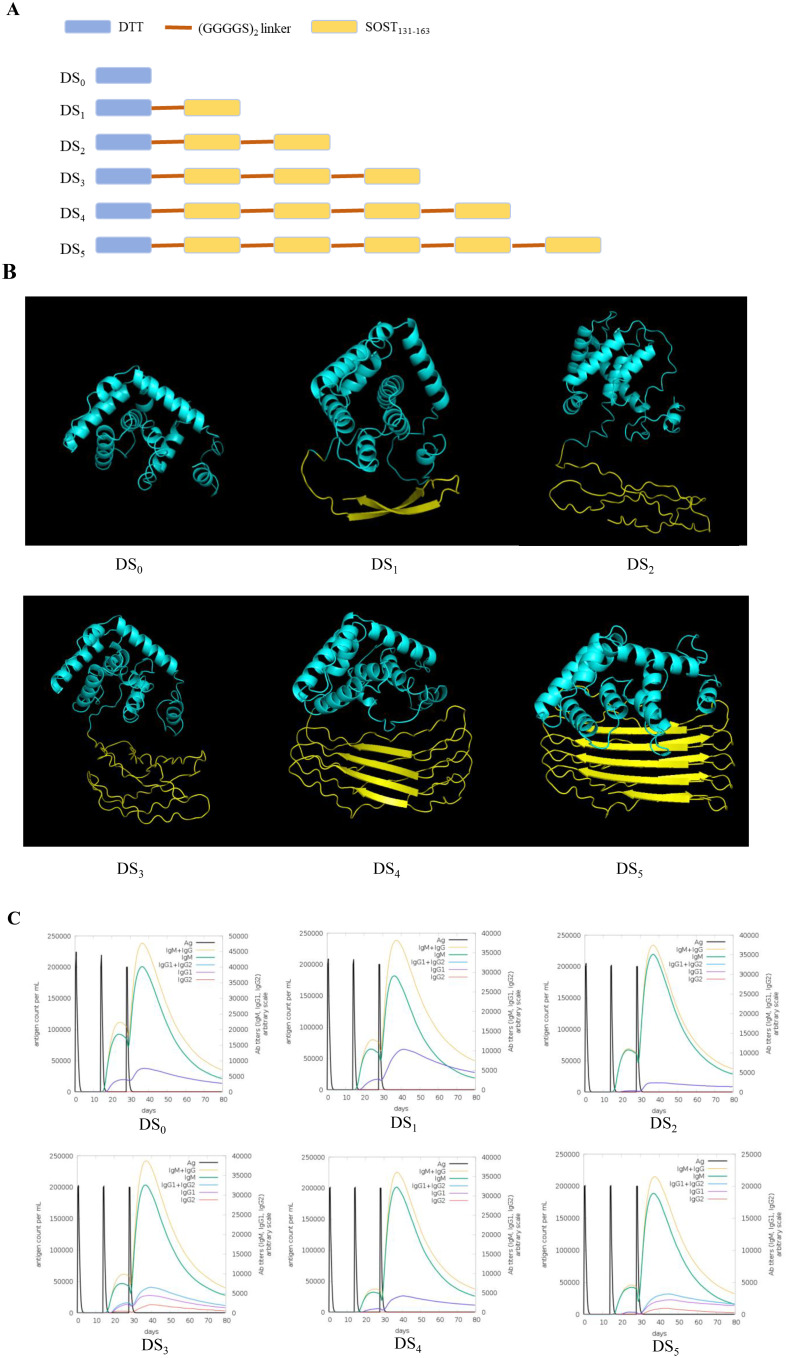
Construction plan and immune stimulation simulation of SOST candidate vaccines. **(A)** Schematic representation for the construction of SOST candidate vaccines. **(B)** Predicted three-dimensional structure of SOST candidate vaccines, modeled using AlphaFold2 server based on amino acid sequence. The cyan region denotes DTT protein scaffold, while the yellow regions represent the various repeated SOST_131–163_ peptides. **(C)** Immune stimulation simulation conducted using C-IMMSIM online server demonstrates that DS_3_ and DS_5_ vaccines simultaneously stimulate the production of IgM, IgG1, and IgG2 (Romosozumab is classified as an IgG2 antibody), whereas other candidate vaccines primarily induced IgM and IgG1 antibodies. Consequently, DS_3_ and DS_5_ vaccines were selected for further analysis.

To enhance vaccine efficacy in inducing antibodies, we conjugated DTT scaffold, which contains substantial T-helper epitopes capable of disrupting immune tolerance to autoantigens, with varying quantities of repetitive SOST_131–163_ epitopes via a (GGGGS)_2_ linker. Six recombinant vaccines were constructed using this method, labeled DS_0_ to DS_5_, corresponding to the incorporation of 0 to 5 copies of SOST_131–163_ epitopes into DTT scaffold (**Figure 2A**). Tertiary structures of these candidate vaccines were predicted using AlphaFold2 server, which generated five structural models for each vaccine. The top-ranked model for each vaccine was selected based on the highest predicted Local Distance Difference Test (pLDDT) score, and the resulting structures are presented (**Figure 2B**).

To evaluate immune-stimulating potential of the top-ranked model for each candidate vaccine, we employed C-IMMSIM online server. The results demonstrated that all vaccine candidates elicited relatively high antibody titers following three immunization injections. Notably, the DS_3_ and DS_5_ vaccines stimulated the production of multiple antibody isotypes, including IgM, IgG1 and IgG2, while other candidate predominantly induced IgM and IgG1 responses (**Figure 2C**). Importantly, the production of the IgG2 subtype is particularly significant as it is the functional antibody associated with ROMO. Since the candidate vaccines are designed to elicit an IgG2 antibody response that closely resembles that of ROMO (21), we selected the DS_3_ and DS_5_ vaccines for further analysis and investigation.

### 3.3 Immunological and physicochemical properties of DS_3_ and DS_5_ vaccines

Safety and efficacy are critical criteria for evaluating vaccines. Analysis using AllerTOP v.2.1 revealed that both DS_3_ and DS_5_ vaccines are non-allergenic. Antigenicity of these vaccines was assessed through VaxiJen v2.0, yielding scores of 0.7434 for DS_3_ and 0.7948 for DS_5_, both surpassing the threshold value of 0.5. These results indicate that DS_3_ and DS_5_ vaccines are not only safe but also exhibit high immunogenicity. Solubility assessments conducted via SolPro server produced favorable scores of 0.713 for DS_3_ and 0.925 for DS_5_. Additional physicochemical parameters were predicted using ExPASy ProtParam server. Both vaccines are classified as recombinant proteins, with molecular weights of 31.8 kDa for DS_3_ and 40.3 kDa for DS_5_, and isoelectric points of 9.14 and 9.61, respectively. The total atom counts were recorded as 4463 for DS_3_ and 5657 for DS_5_. Estimated half-lives for both vaccines are approximately 30 hours in mammalian reticulocytes, over 20 hours in yeast, and exceeding 10 hours in Escherichia coli. The instability indices were calculated to be 42.19 for DS_3_ and 39.93 for DS_5_, while aliphatic indices measured 82.72 and 79.38, respectively. The grand average of hydropathicity (GRAVY) values were -0.257 for DS_3_ and -0.294 for DS_5_ (**Table 1**).

**Table 1:** Prediction of immunological and physicochemical properties for DS_3_ and DS_5_ vaccines.

Property	DS_3_	DS_5_
Allergenicity(AllerTOP v2.1)	NON-ALLERGEN	NON-ALLERGEN
Antigenicity (VaxiJen v2.0)	0.7434	0.7948
Solubility (SOLpro)	0.713771	0.925892
Number of amino acids	302	386
Molecular weight	31804.32	40334.16
Theoretical Isoelectric point (pI)	9.14	9.61
Formula	C_1362_H_2240_N_424_O_421_S_16_	C_1716_H_2840_N_556_O_523_S_22_
Total number of atoms	4463	5657
Estimated half-life	30 hours (mammalian reticulocytes, *in vitro*). >20 hours (yeast, *in vivo*). >10 hours (Escherichia coli, *in vivo*).	30 hours (mammalian reticulocytes, *in vitro*). >20 hours (yeast, *in vivo*). >10 hours (Escherichia coli, *in vivo*)
Instability index	42.19(unstable)	39.93(stable)
Aliphatic index	82.72	79.38
Grand average of hydropathicity (GRAVY)	-0.257	-0.294

### 3.4 Assessment of secondary structure of DS_3_ and DS_5_ vaccines

**Figure 3:**
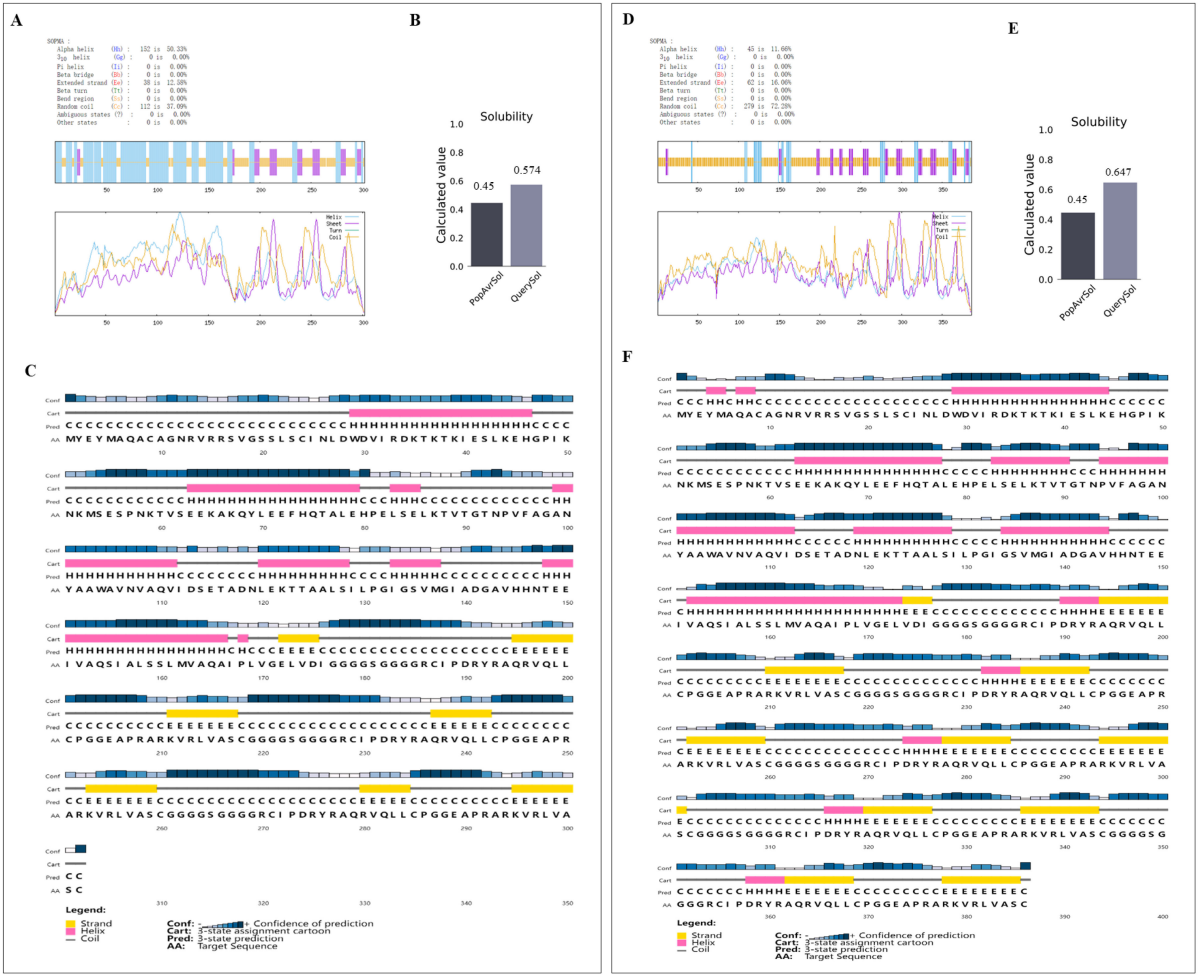
Analysis of the secondary structure and solubility characteristics of DS_3_ and DS_5_ vaccine. Secondary structure of DS_3_**(A)** and DS_5_**(D)** was assessed using SOPMA server. Solubility characteristics of DS_3_**(B)** and DS_5_**(E)** vaccines were evaluated using Protein-Sol server, resulting in solubility scores of 0.574 and 0.647, respectively, both exceeding the baseline value of 0.45, indicating enhanced solubility. Secondary structure analysis of DS_3_**(C)** and DS_5_**(F)** was performed using PESIPRED web server, the blue bars show the confidence of prediction.

Secondary structure compositions of DS_3_ and DS_5_ vaccine were analyzed using SOPMA server. DS_3_ candidate exhibited a secondary structure composed of 50.33% α-helices (152/302), 37.09% random coils (112/302), and 12.58% extended strands (38/302) (**Figure 3A**). Additionally, DS_3_ demonstrated enhanced solubility, with a Protein-Sol score of 0.574, surpassing the baseline threshold of 0.45 (**Figure 3B**). Confidence in the secondary structure predictions for DS_3_ was further assessed using the PESIPRED web server, which provided favorable results (**Figure 3C**). In contrast, DS_5_ candidate displayed a distinct secondary structure profile, characterized by 11.66% α-helices (45/386), 72.28% random coils (279/386), and 16.06% extended strands (62/386) (**Figure 3D**). Similarly, DS_5_ exhibited enhanced solubility, with a Protein-Sol score of 0.647, exceeding the baseline value of 0.45 (**Figure 3E**). Confidence of the secondary structure predictions for DS_5_ was also assessed using PESIPRED web server, yielding favorable results (**Figure 3F**).

### 3.5 Analysis and refinement of tertiary structures of DS_3_ and DS_5_ vaccine

Top-ranked models for DS_3_ and DS_5_ vaccines, predicted using AlphaFold2, exhibited pLDDT scores of 37.1 and 45.1, respectively. The pLDDT scores, which range from 0 to 100, serve as an indicator of model confidence, where values above 80 reflect high confidence in the accuracy of residue structure, and scores below 50 suggest the presence of disordered regions. Both DS_3_ and DS_5_ displayed pLDDT scores below the confidence threshold of 50, warranting further refinement.

**Figure 4:**
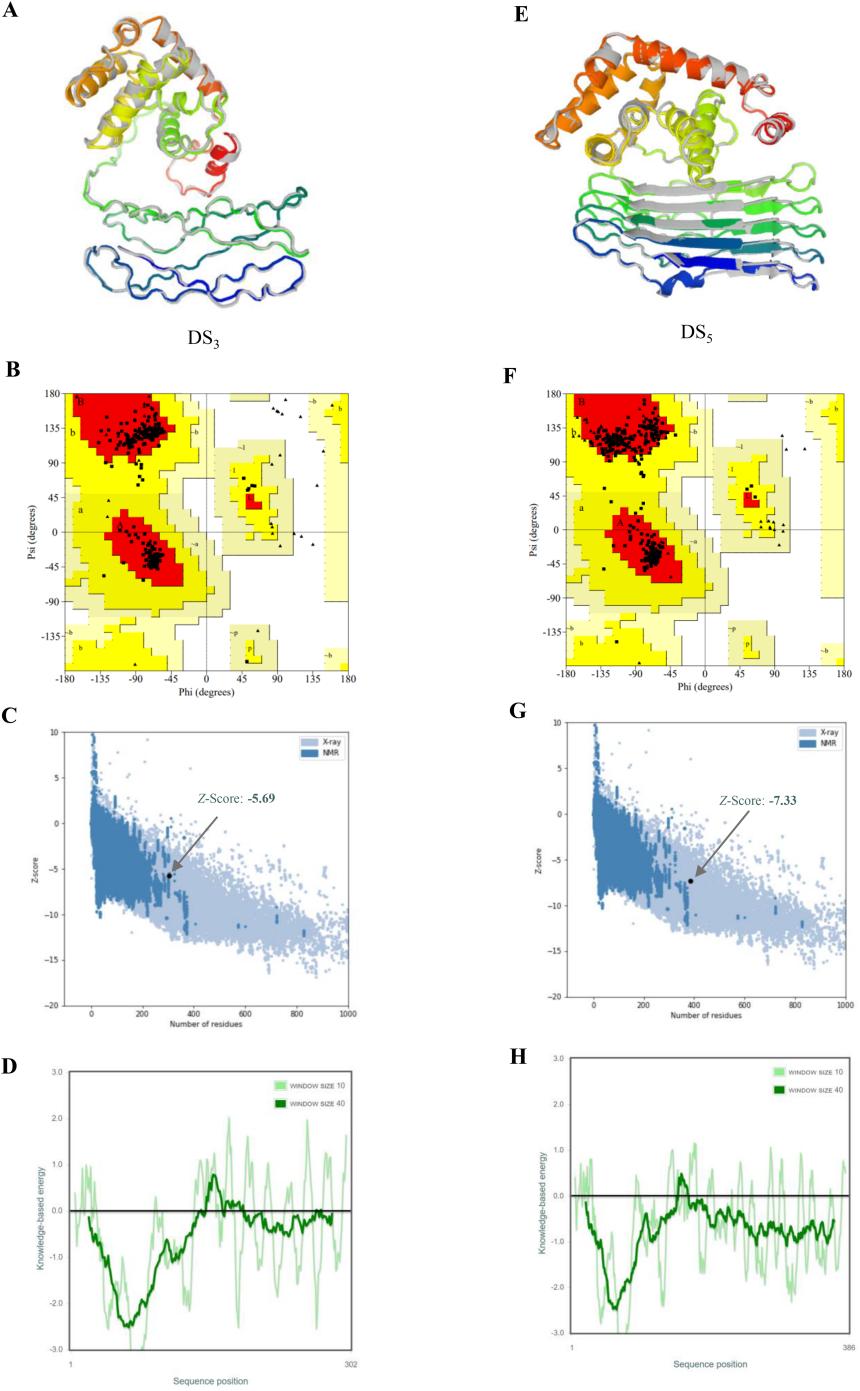
Refinement and validation of tertiary structures for DS_3_ and DS_5_ vaccines. Tertiary structures of DS_3_**(A)** and DS_5_**(E)** were refined using GalaxyRefine web server, with initial structures depicted in gray and refined structures shown in rainbow colors. Ramachandran plots for refined structures, generated via PROCHECK, indicate that 94.3% of DS_3_ residues **(B)** are located in the most favored regions, 5.7% in additional allowed regions, 0.0% in generously allowed regions, and 0.0% in disallowed regions. For DS_5_**(F)**, 93.7% of residues are in the most favored regions, 6.3% in additional allowed regions, and 0.0% in both generously allowed and disallowed. Z-scores obtained from ProSA-web for DS_3_**(C)** and DS_5_**(G)** models are -5.69 and -7.33, respectively (black dots), both within the conformational score range for experimentally validated protein structures. Panels **(D)** and **(H)** display energy plots of the amino acid compositions for DS_3_ and DS_5_, respectively.

Refinement was conducted using GalaxyRefine server, resulting in the generation of five refined models for each vaccine candidate. Optimal model quality is characterized by higher Global Distance Test High Accuracy (GDT-HA) and Ramachandran values, and lower root-mean-square deviation (RMSD), MolProbity scores, clash scores, and counts of poor rotamers. For DS3, Model 1 demonstrated the most favorable refinement metrics, achieving GDT-HA of 0.9007, RMSD of 0.580 Å, MolProbity score of 1.459, clash score of 3.7, poor rotamer count of 0.4, and Ramachandran favored percentage of 95.7% (**Figure 4A**, **Supplementary Table S1**). Similarly, Model 1 of DS5 exhibited optimal refinement results, with GDT-HA of 0.9424, RMSD of 0.480 Å, MolProbity score of 1.772, clash score of 10.2, poor rotamer count of 0.0, and Ramachandran favored of 96.4% (**Figure 4E**, **Supplementary Table S2**).

Ramachandran plots generated via PROCHECK indicated that 94.3% of DS_3_ residues reside in the most favored regions, with 5.7% in additional allowed regions, while no residues were found in generously allowed or disallowed regions (**Figure 4B**). Conversely, for DS_5_, 93.7% of residues were in the most favored regions, 6.3% in additional allowed regions, with none in generously allowed or disallowed regions (**Figure 4F**). The Z-scores calculated from ProSA-web were -5.69 for DS_3_ model and -7.33 for DS_5_ model (**Figures 4C, G**), both of which fall within the acceptable range for conformational scores typical of experimentally validated protein structures. DS_3_ vaccine exhibited a quality factor of 90.8451 according to ERRAT, while DS_5_ vaccine received a quality factor of 63.2867. Furthermore, energy plots corresponding to the amino acid compositions for both DS_3_ (**Figure 4D**) and DS_5_ (**Figure 4H**) were analyzed, providing additional evidence for structural integrity of the refined models.

### 3.6 T-cell and B-cell epitopes of DS_3_ and DS_5_ vaccine

**Figure 5:**
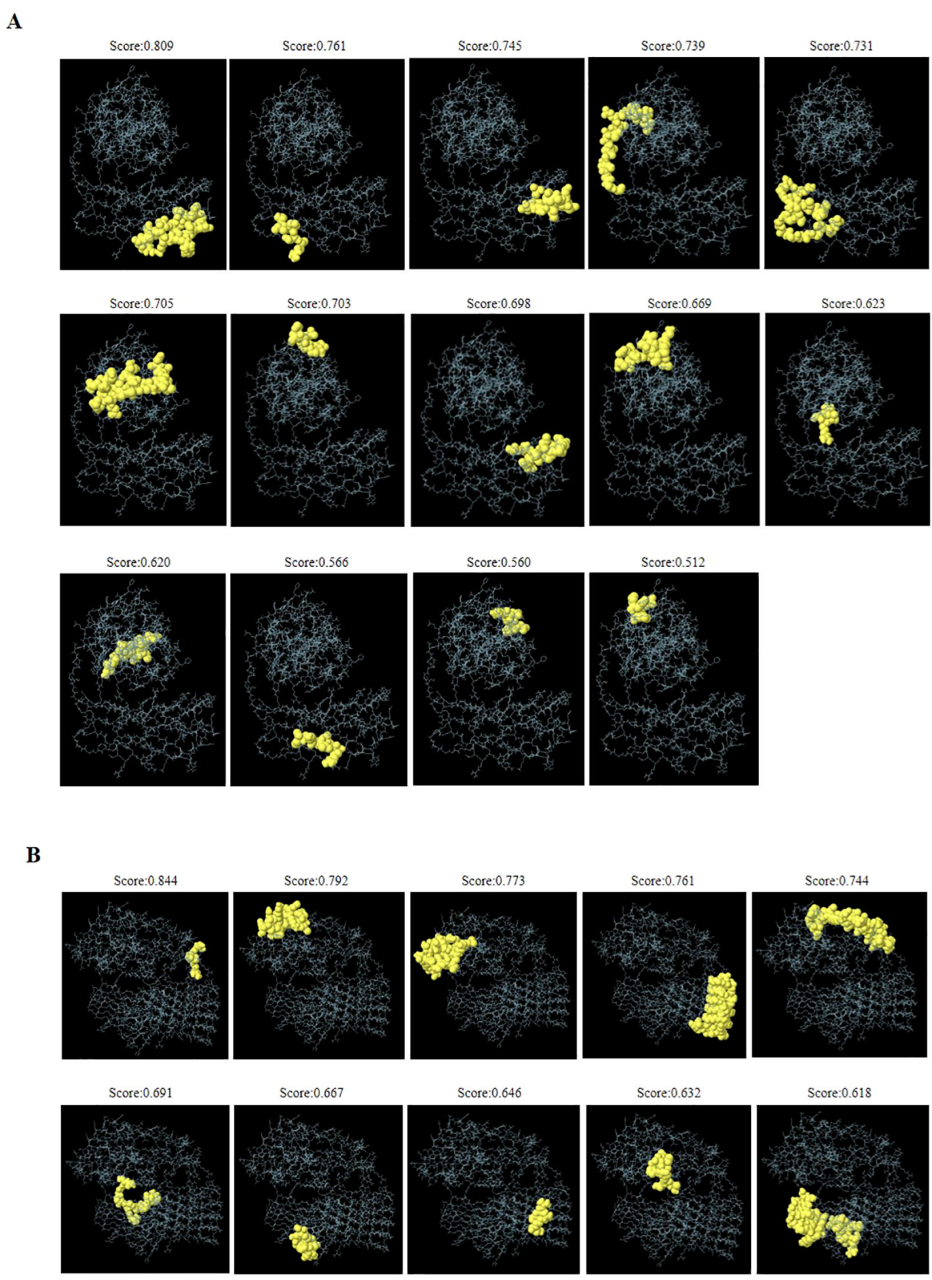
Three-dimensional representation of discontinuous B-cell epitopes predicted for the refined DS_3_ and DS_5_ vaccines. **(A)** Fourteen discontinuous B-cell epitopes of the refined DS_3_ vaccine are displayed, while **(B)** ten discontinuous B-cell epitopes of the refined DS_5_ vaccine are shown. The discontinuous B-cell epitopes are represented as yellow spheres, with the remaining vaccine residues illustrated as gray sticks.

Analysis using IEDB reveals that DS_3_ vaccine exhibits a high density of T-cell epitopes, comprising 13 strong and 34 weak CTL binders, as well as 3 strong and 47 weak HTL binders (**Supplementary Table S3**). Additionally, the ElliPro tool identifies 14 conformational B-cell epitopes associated with DS_3_ vaccine (**Figure 5A**, **Table 2**). In contrast, DS_5_ vaccine also demonstrates a rich repertoire of T-cell epitopes, featuring 17 strong and 42 weak CTL binders, along with 5 strong and 55 weak HTL binders (**Supplementary Table S3**). Moreover, a total of 10 conformational B-cell epitopes are predicted for DS_5_ vaccine, as indicated by ElliPro (**Figure 5B**, **Table 2**).

**Table 2:** Predicted discontinuous B-cell epitopes of the refined DS_3_ and DS_5_ vaccines.

Vaccine	No.	Residues	No. of residues	Score
DS_3_	1	A:R277, A:A278, A:Q279, A:R280, A:V281, A:Q282, A:L283, A:L284, A:C285, A:P286, A:G287, A:G288, A:E289, A:A290, A:P291, A:R292, A:A293, A:R294, A:K295, A:V296	20	0.809
	2	A:C271, A:I272, A:P273, A:D274, A:R275	5	0.761
	3	A:V197, A:Q198, A:L199, A:L200, A:C201, A:P202, A:G203, A:G204, A:E205, A:A206, A:P207, A:A209	12	0.745
	4	A:I167, A:P168, A:L169, A:V170, A:G171, A:E172, A:L173, A:V174, A:D175, A:I176, A:G177, A:G178, A:G179, A:G180, A:S181, A:G182, A:G183, A:G184	18	0.739
	5	A:G220, A:G221, A:G222, A:S223, A:G224, A:G225, A:G226, A:G227, A:R228, A:C229, A:I230, A:P231, A:D232, A:A258, A:S259, A:C260, A:G261, A:G262, A:G263, A:G264, A:S265, A:G266, A:G267, A:G268, A:G269, A:R270	26	0.731
	6	A:S56, A:P57, A:N58, A:K59, A:T60, A:V61, A:S62, A:E63, A:E64, A:K65, A:A66, A:K67, A:Q68, A:Y69, A:D113, A:S114, A:E115, A:T116, A:A117, A:D118, A:N119	21	0.705
	7	A:T38, A:E41, A:S42, A:K44, A:E45, A:H46	6	0.703
	8	A:R238, A:L241, A:L242, A:C243, A:P244, A:G245, A:G246, A:E247, A:A248, A:P249, A:A251, A:R252	12	0.698
	9	A:G47, A:P48, A:K50, A:N51, A:K52, A:M53, A:S54, A:E55, A:Q75, A:T76, A:E79	11	0.669
	10	A:V14, A:R15, A:R16	3	0.623
	11	A:S17, A:V18, A:G19, A:S20, A:S21, A:L22, A:S23, A:C24, A:I25, A:N26, A:L27	11	0.62
	12	A:R297, A:L298, A:V299, A:A300, A:S301, A:C302	6	0.566
	13	A:D28, A:D30, A:V31, A:D34, A:K35	5	0.56
	14	A:H80, A:P81, A:E82, A:L83	4	0.512
DS_5_	1	A:V14, A:R15, A:R16	3	0.844
	2	A:I40, A:E41, A:S42, A:L43, A:K44, A:E45, A:H46, A:G47, A:P48, A:I49, A:K50, A:N51, A:K52, A:M53, A:S54, A:E55, A:Q75, A:T76, A:E79	19	0.792
	3	A:S56, A:P57, A:N58, A:K59, A:T60, A:V61, A:S62, A:E63, A:E64, A:K65, A:A66, A:Q68, A:Y69, A:E72, A:I112, A:D113, A:S114, A:E115, A:T116, A:A117, A:D118, A:N119, A:L120, A:K122	24	0.773
	4	A:L200, A:C201, A:P202, A:G203, A:G204, A:E205, A:A206, A:P207, A:R208, A:L242, A:C243, A:P244, A:G245, A:G246, A:E247, A:A248, A:P249, A:R250, A:L284, A:C285, A:P286, A:G287, A:G288, A:E289, A:A290, A:P291, A:R292, A:L326, A:C327, A:P328, A:G329, A:G330, A:E331, A:A332, A:P333, A:R334, A:Q366, A:L367, A:L368, A:C369, A:P370, A:G371, A:G372, A:E373, A:A374, A:P375, A:R376	47	0.761
	5	A:A6, A:C9, A:A10, A:G11, A:N12, A:S17, A:V18, A:G19, A:S20, A:S21, A:L22, A:S23, A:C24, A:I25, A:N26, A:L27, A:D28, A:D30, A:V31, A:I32, A:D34, A:K35, A:K37, A:T38, A:K39, A:H80, A:P81, A:E82, A:L83, A:S84, A:K87	31	0.744
	6	A:G180, A:S181, A:G182, A:G183, A:G184, A:G185, A:R186, A:C187, A:I188, A:P189, A:D190, A:R191	12	0.691
	7	A:I314, A:P315, A:D316, A:C355, A:I356, A:P357, A:D358, A:R359, A:C386	9	0.667
	8	A:R336, A:A377, A:R378	3	0.646
	9	A:A140, A:D141, A:G142, A:A143, A:V144, A:H145, A:H146, A:N147, A:T148	9	0.632
	10	A:G222, A:S223, A:G224, A:G225, A:G226, A:G227, A:R228, A:I230, A:P231, A:D232, A:G264, A:S265, A:G266, A:G267, A:G268, A:G269, A:R270, A:P273, A:D274, A:R275, A:R277, A:G306, A:S307, A:G308, A:G309, A:G310, A:G311, A:R312, A:R317, A:R319, A:G347, A:G348, A:S349, A:G350, A:G351, A:G352, A:G353, A:R354	38	0.618

### 3.7 Molecular docking and molecular dynamics simulation

**Figure 6:**
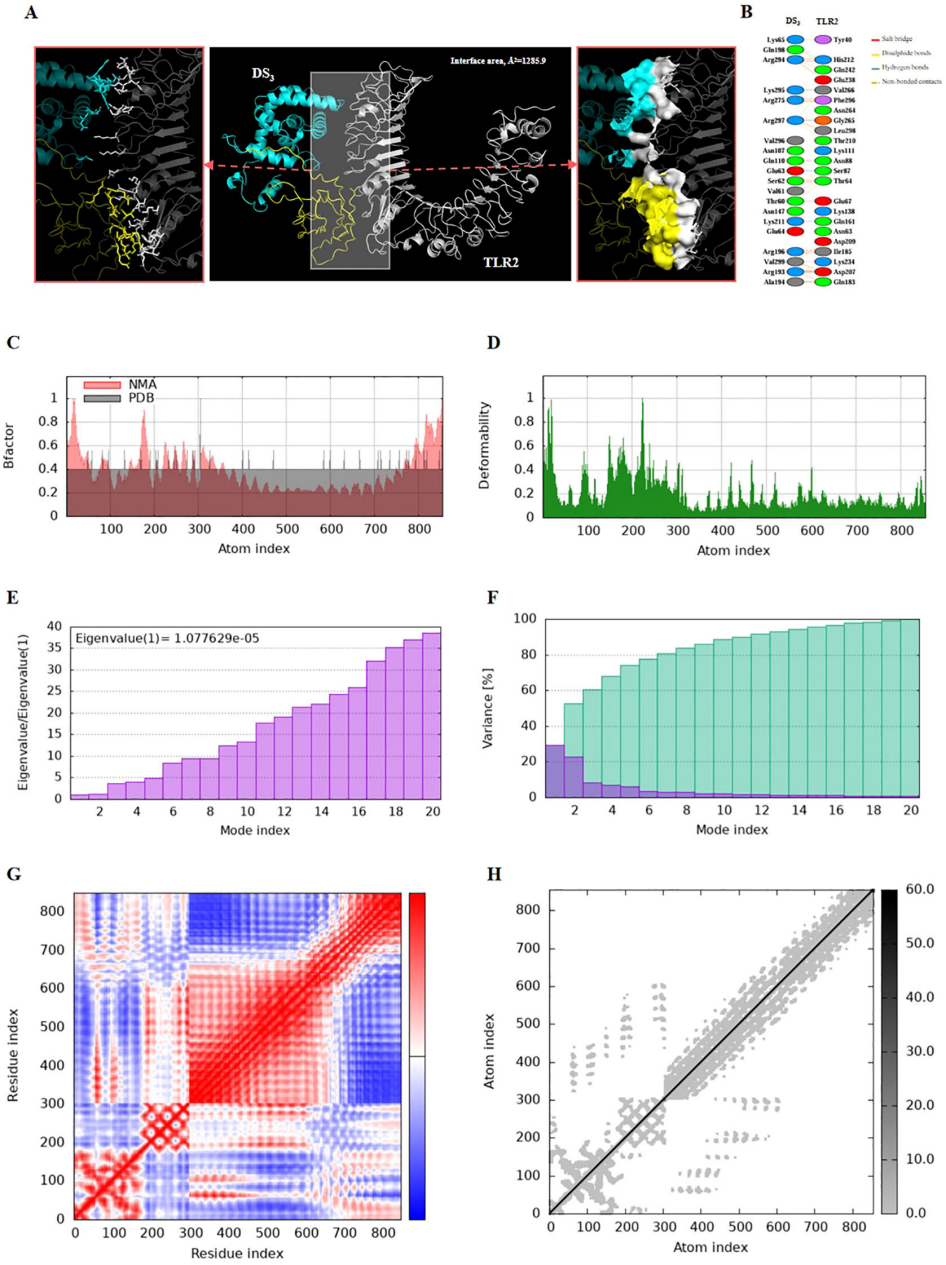
Molecular docking and normal mode analysis of DS_3_ with TLR2. **(A)** 3D model of the DS_3_ –TLR2 docking complex illustrating their interactions; DS_3_ is colored cyan-yellow and TLR2 is shown in white. Overall structures are represented as cartoons, with key interface residues emphasized. Potential interactions are depicted as sticks and surface. The measured binding free energy is -46.21 kcal/mol, with an interface area of 1285.9 Å². **(B)** Detailed interactions between DS_3_ and TLR2, including 1 salt bridges (red), 6 hydrogen bonds (blue), and 137 non-bonded contacts (yellow-orange). **(C)** B-factor representation of the docking complex. **(D)** Deformability plot of the complex. **(E)** Eigenvalues associated with the docked complex. **(F)** Variance analysis of the docked complex. **(G)** Covariance map of atomic pairs of amino acid residues; correlated interactions are shown in red, uncorrelated in white, and anti-correlated in blue. **(H)** Elastic network model of the docking complex, with darker gray indicating stiffer springs.

**Figure 7:**
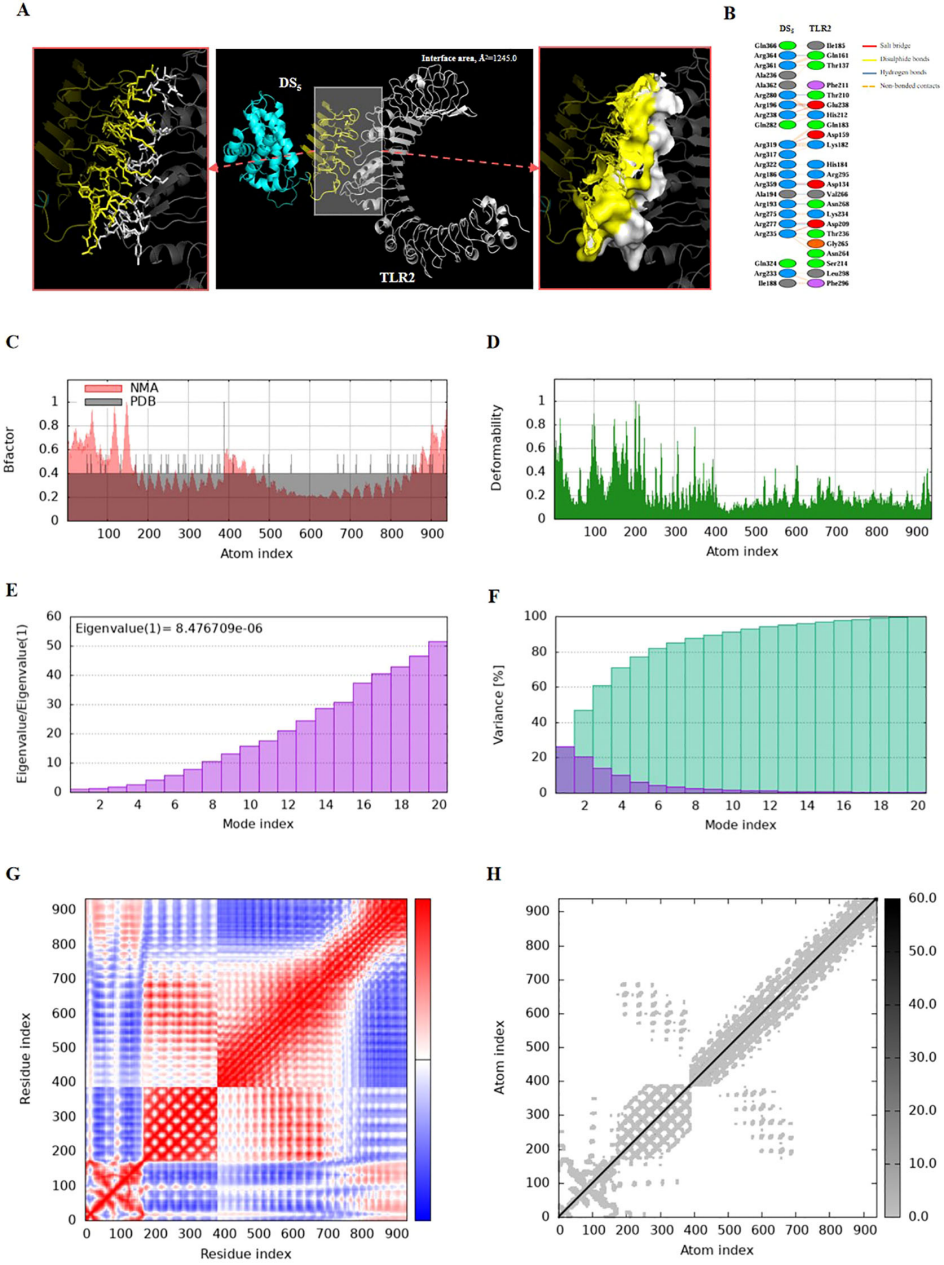
Molecular docking and normal mode analysis of DS_5_ with TLR2. **(A)** 3D model of the DS_5_ –TLR2 docking complex illustrating their interactions; DS_3_ is colored cyan-yellow and TLR2 is shown in white. Overall structures are represented as cartoons, with key interface residues emphasized. Potential interactions are depicted as sticks and surface. The measured binding free energy is -79.05 kcal/mol, with an interface area of 1245.0 Å². **(B)** Detailed interactions between DS_5_ and TLR2, including 6 salt bridges (red), 7 hydrogen bonds (blue), and 142 non-bonded contacts (yellow-orange). **(C)** B-factor representation of the docking complex. **(D)** Deformability plot of the complex. **(E)** Eigenvalues associated with the docked complex. **(F)** Variance analysis of the docked complex. **(G)** Covariance map of atomic pairs of amino acid residues; correlated interactions are shown in red, uncorrelated in white, and anti-correlated in blue. **(H)** Elastic network model of the docking complex, with darker gray indicating stiffer springs.

Molecular docking and molecular dynamics simulations were performed to elucidate the interactions between the DS_3_ and DS_5_ vaccines and Toll-like receptor 2 (TLR2). For molecular docking analysis, HawkDock was conducted to generate ten docking models for each vaccine. The optimized docking models revealed binding scores of -6618.72 kcal/mol for DS_3_ and -8177.20 kcal/mol for DS_5_, suggesting a stronger interaction between DS_5_ and TLR2. Further structural analysis of DS_3_-TLR2 and DS_5_-TLR2 complexes indicated distinct binding energies and interface areas. Specifically, DS_3_-TLR2 complex demonstrated a binding energy of -46.21 kcal/mol with an interface area of 1285.9 Å² (**Figure 6A**), whereas DS_5_-TLR2 complex exhibited a higher binding energy of -79.05 kcal/mol and a surface area of 1245.0 Å² (**Figure 7A**). DS_3_-TLR2 complex was characterized by the presence of 1 salt bridge, 6 hydrogen bonds, and 137 non-bonded contacts (**Figure 6B**), while DS_5_-TLR2 complex featured 6 salt bridges, 7 hydrogen bonds, and 142 non-bonded contacts (**Figure 7B**).

Both complexes demonstrated stability, as evidenced by their B-factors (**Figures 6C**, **7C**), deformability profiles (**Figures 6D**, **7D**), and low eigenvalues (8.476709e-06 for DS_5_-TLR2 and 1.077629e-05 for DS_3_-TLR2) (**Figures 6E**, **7E**), as well as variance analyses (**Figures 6F**, **7F**). Covariance matrix evaluations highlighted correlations, as well as uncorrelated and anti-correlated motions among the residues within the complexes (**Figures 6G**, **7G**). Elastic network analysis illustrated spring-like interactions between atoms, with darker gray depicting stiffer springs (**Figures 6H**, **7H**). Collectively, these findings suggest that both DS_3_ and DS_5_ vaccines effectively engage TLR2, potentially eliciting robust immune responses.

### 3.8 Immune response simulation induced by vaccines

**Figure 8:**
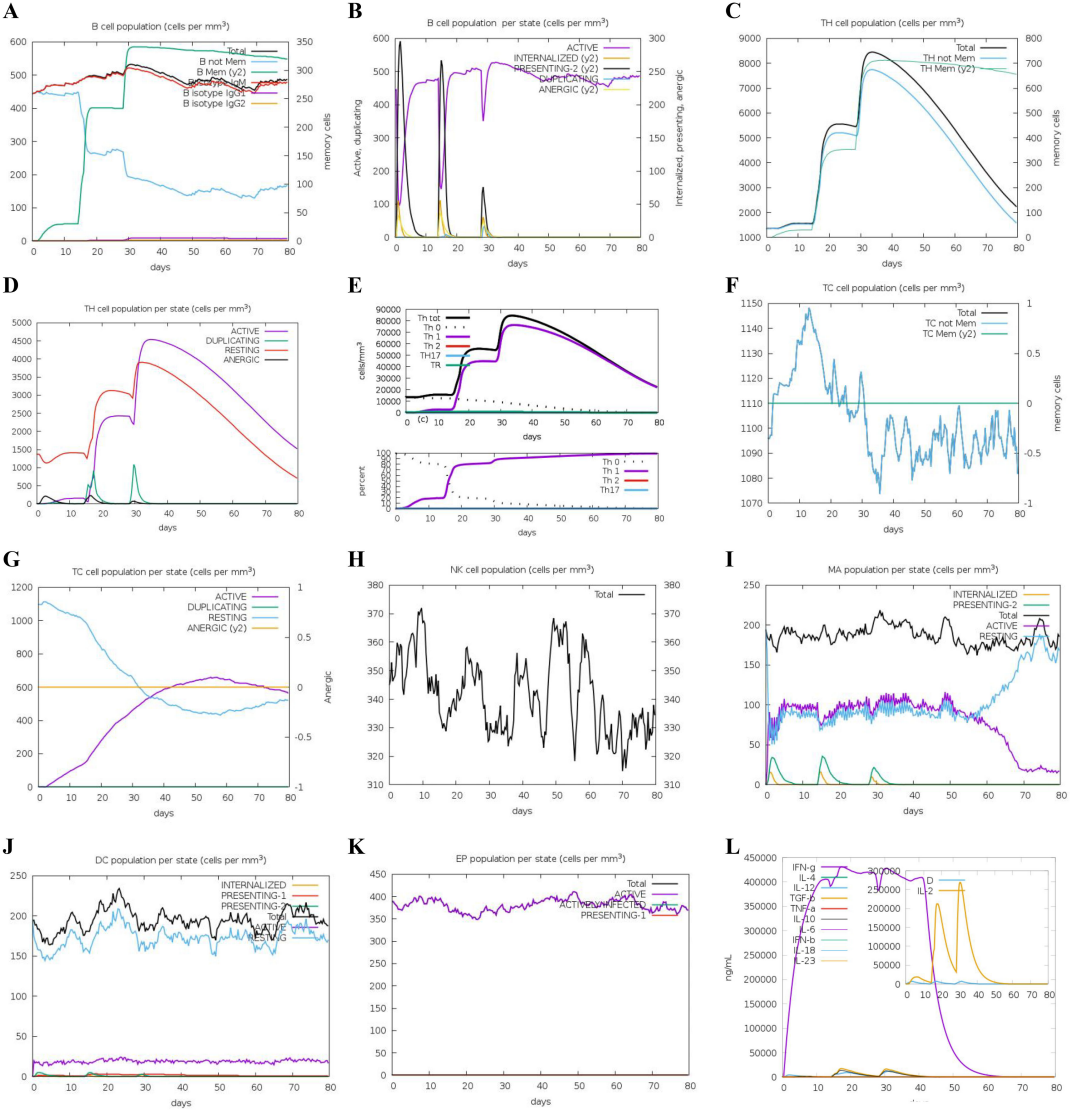
Predicted immune response induced by three administrations of DS_3_ vaccine via C-IMMSIM online server. Vaccinations were conducted on Day 1, Day 14, and Day 28. **(A)** Changes in B cell populations after vaccination, with specific subclasses color-coded. **(B)** Levels of B cell production post-immunization; active B cells (depicted in purple) show the highest secretion among subtypes. **(C)** Production of CD4+ T-helper (TH) cells in response to antigen exposure. **(D)** Distribution of TH cell states, including active, duplicating, resting, and anergic cells. **(E)** Quantification and proportion of different TH cell subtypes. **(F)** Levels of cytotoxic T (TC) cell production. **(G)** Overview of the TC cell population, categorized into resting and active states over time after DS_3_ vaccination. **(H)** Distribution of natural killer (NK) cells. **(I)** States of macrophages (MA). **(J)** Status of dendritic **(DC)** cells. **(K)** Production levels of epithelial cells. **(L)** Cytokine levels following DS_3_ vaccination. The main plot depicts overall cytokine concentrations, while the inset illustrates the levels of danger signals alongside the leukocyte growth factor IL-2.

**Figure 9:**
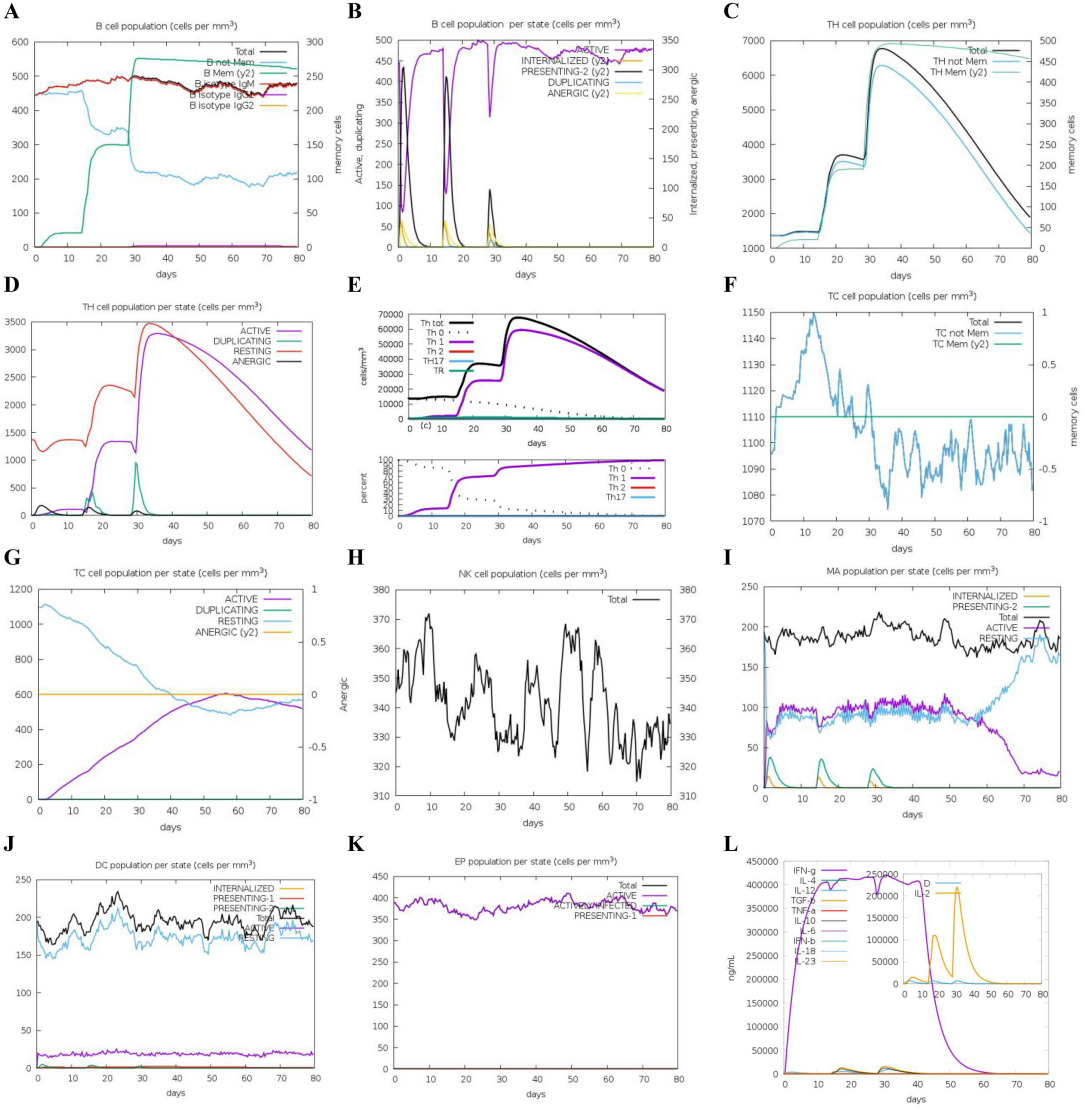
Predicted immune response induced by three administrations of DS_5_ vaccine via C-IMMSIM online server. Vaccinations were conducted on Day 1, Day 14, and Day 28. **(A)** Changes in B cell populations after vaccination, with specific subclasses color-coded. **(B)** Levels of B cell production post-immunization; active B cells (depicted in purple) show the highest secretion among subtypes. **(C)** Production of CD4+ T-helper (TH) cells in response to antigen exposure. **(D)** Distribution of TH cell states, including active, duplicating, resting, and anergic cells. **(E)** Quantification and proportion of different TH cell subtypes. **(F)** Levels of cytotoxic T (TC) cell production. **(G)** Overview of the TC cell population, categorized into resting and active states over time after DS_5_ vaccination. **(H)** Distribution of natural killer (NK) cells. **(I)** States of macrophages (MA). **(J)** Status of dendritic **(DC)** cells. **(K)** Production levels of epithelial cells. **(L)** Cytokine levels following DS_5_ vaccination. The main plot depicts overall cytokine concentrations, while the inset plot shows danger signal together with leukocyte growth factor IL-2.

To assess the adaptive immune responses elicited by DS_3_ and DS_5_ vaccines, we employed C-IMMSIM server to simulate *in vivo* immune reactions. Our analysis revealed an increase in the total B cell population, including B-memory cells and IgM isotypes, which contributed to a significant rise in activated B cells in the host (**Figures 8**, **9A, B**). Following the second immunization, the total count of T helper (TH) cells exhibited a rapid increase, peaking after the third immunization (**Figures 8**, **9C**). Both activated and resting TH cell populations surged after each injection, primarily comprising TH1 cells (**Figures 8**, **9D, E**), suggesting effective antibody maturation processes. Notably, the population of anergic (y2) T cells remained stable throughout the duration of the study (**Figures 8**, **9F**). In contrast, the count of activated T cytotoxic (TC) cells showed a transient increase followed by a decline, while resting TC cells exhibited the opposite trend (**Figures 8**, **9G**).

Additionally, we evaluated the effects of DS_3_ and DS_5_ vaccines on innate immune cell populations. Natural killer (NK) cells, dendritic cells (DCs), and active epithelial cell populations demonstrated a relatively stable response upon immunization (**Figures 8**, **9H, J, K**). Upon initial immunization, there was a marked increase in both active and resting macrophage populations in a short time, which subsequently reached a peak and stabilized (**Figures 8**, **9I**). Approximately four weeks after the third immunization, we observed a decline in the number of active macrophages, coinciding with a rapid increase in resting macrophages (**Figures 8**, **9I**). Following the administration of DS_3_ and DS_5_ vaccines, there was an activation of downstream inflammatory mediators, with significant elevations in levels of IFN-γ and IL-2 (**Figures 8**, **9L**). Collectively, these findings indicated that the DS_3_ and DS_5_ vaccines effectively stimulate both innate and adaptive immune responses, highlighting their potential as effective vaccine candidates.

### 3.9 Cloning, expression and immunogenicity of vaccines

**Figure 10:**
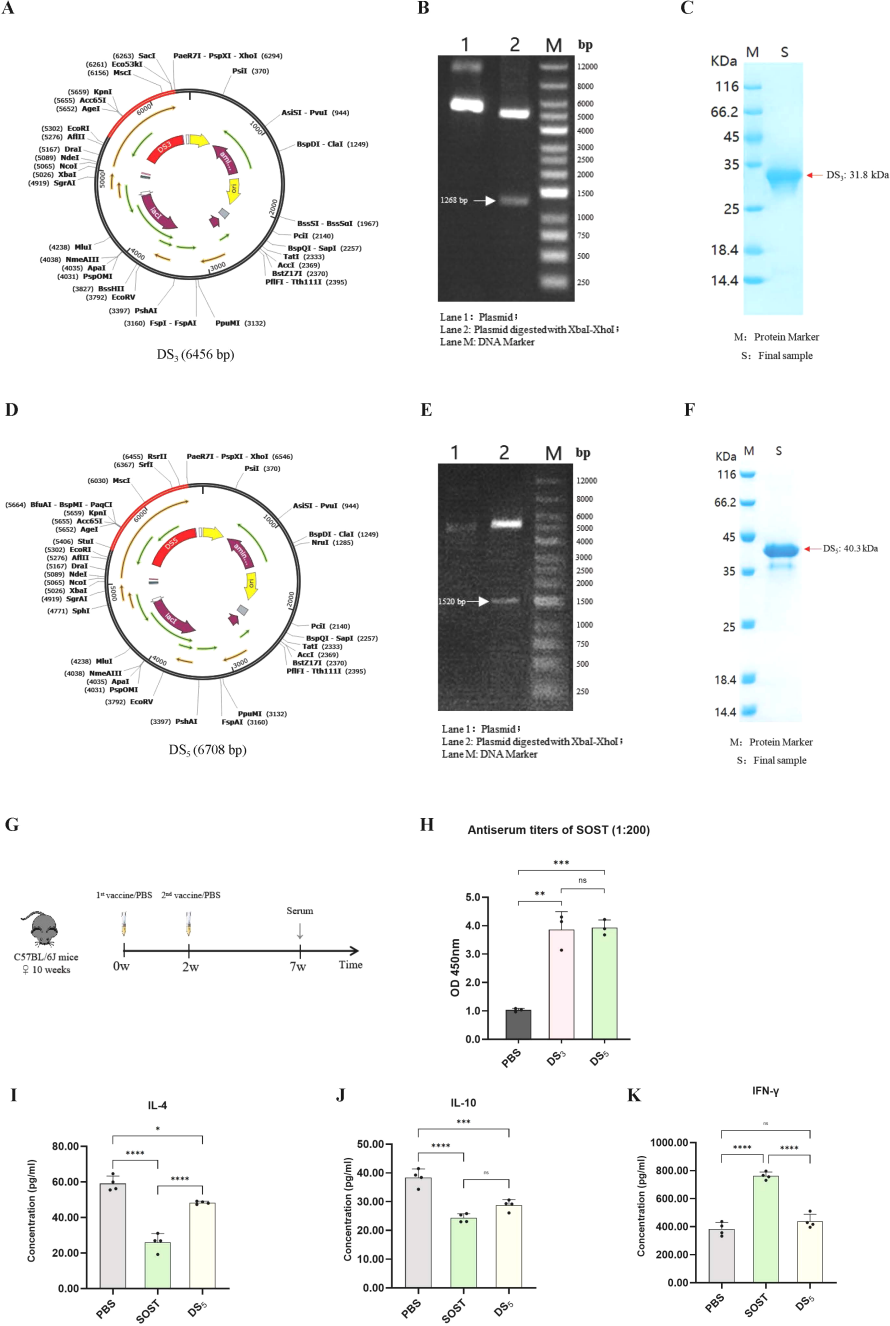
Cloning, expression, and immunogenicity assessment of DS_3_ and DS_5_ vaccines. **(A)** DS_3_ vaccine sequence (red) was inserted into the pSmartI expression vector (black) via seamless cloning using XhoI. **(B)** Agarose gel electrophoresis showing: Lane 1, recombinant plasmid; Lane 2, target fragment (1268 bp) along with vector sequence; Lane M, 1 kb DNA ladder. **(C)** Expression and purification of DS_3_ vaccine. **(D)** DS_5_ vaccine sequence (red) was cloned into the pSmartI expression vector (black) via seamless cloning using XhoI. **(E)** Agarose gel electrophoresis illustrating: Lane 1, recombinant plasmid; Lane 2, target fragment (1520 bp) along with vector sequence; Lane M, 1 kb DNA ladder. **(F)** Expression and purification of DS_5_. **(G)** Schematic overview of the mouse immunization protocol; each group comprised three mice (n=3), serving as independent biological replicates. **(H)** ELISA measurements indicating significantly elevated serum titers of anti-SOST antibodies in mice immunized with DS_3_ and DS_5_ compared to PBS controls (serum dilution 1:200). Antibody assays were performed in technical duplicates per mouse. **(I–K)** Cytokine levels of IL-4, IL-10, and IFN-γ in supernatants from splenocyte stimulation assays. Data are expressed as mean ± SD. Statistical significance was determined by one-way ANOVA followed by Tukey’s multiple comparisons test. (^*^p < 0.05, ^**^P < 0.01, ^***^P < 0.001, ^****^P < 0.0001, ns = no significance).

Codons for the optimized DS_3_ and DS_5_ sequences were successfully cloned into pSmartI plasmids at XhoI restriction sites (**Figures 10A, D**). The recombinant plasmids were confirmed through PCR amplification, as shown by agarose gel electrophoresis (**Figures 10B, E**). Subsequently, the recombinant plasmids were transformed into Escherichia coli BL21(DE3), leading to the successful expression and purification of DS_3_ and DS_5_ vaccine proteins, which exhibited molecular weights of 31.8 kDa and 40.3 kDa, respectively (**Figures 10C, F**).

To evaluate immunogenic potential of DS_3_ and DS_5_ vaccines, mice were immunized with DS_3_ and DS_5_ proteins at a dose of 200 µg per mouse, with a two-week interval between doses (n=3). Serum was collected at the seventh week to assess antibody production against SOST (**Figure 10G**). Our results demonstrated that both DS_3_ and DS_5_ effectively elicited a significant immune response, resulting in the production of high titers of anti-SOST antibodies in the immunized mice. The antibody titers induced by both vaccines were significantly higher than those observed in PBS control group (**Figures 10H**).

To assess T cell responses to vaccine stimulation, cytokine production associated with Th1 and Th2 responses was quantified using DS_5_ as a representative antigen. Specifically, levels of IL-4 and IL-10 (Th2 markers) and IFN-γ (Th1 marker) were measured in splenocyte cultures stimulated with PBS, SOST, or DS_5_ (**Figures 10I–K**). IFN-γ levels did not differ significantly among the PBS and DS_5_ groups, indicating that DS_5_ does not elicit a robust Th1-mediated cytotoxic response (**Figure 10K**). In contrast, IL-4 and IL-10 secretion were reduced following stimulation with both SOST and DS_5_ (**Figures 10I, J**). Notably, cytokine levels in DS_5_-treated splenocytes remained higher than in SOST-treated cells, suggesting that the DS_5_ vaccine induces a moderated Th2 response that may support B cell-mediated anti-SOST antibody production.

### 3.10 Validation of anti-SOST antiserum function *in vitro*

**Figure 11:**
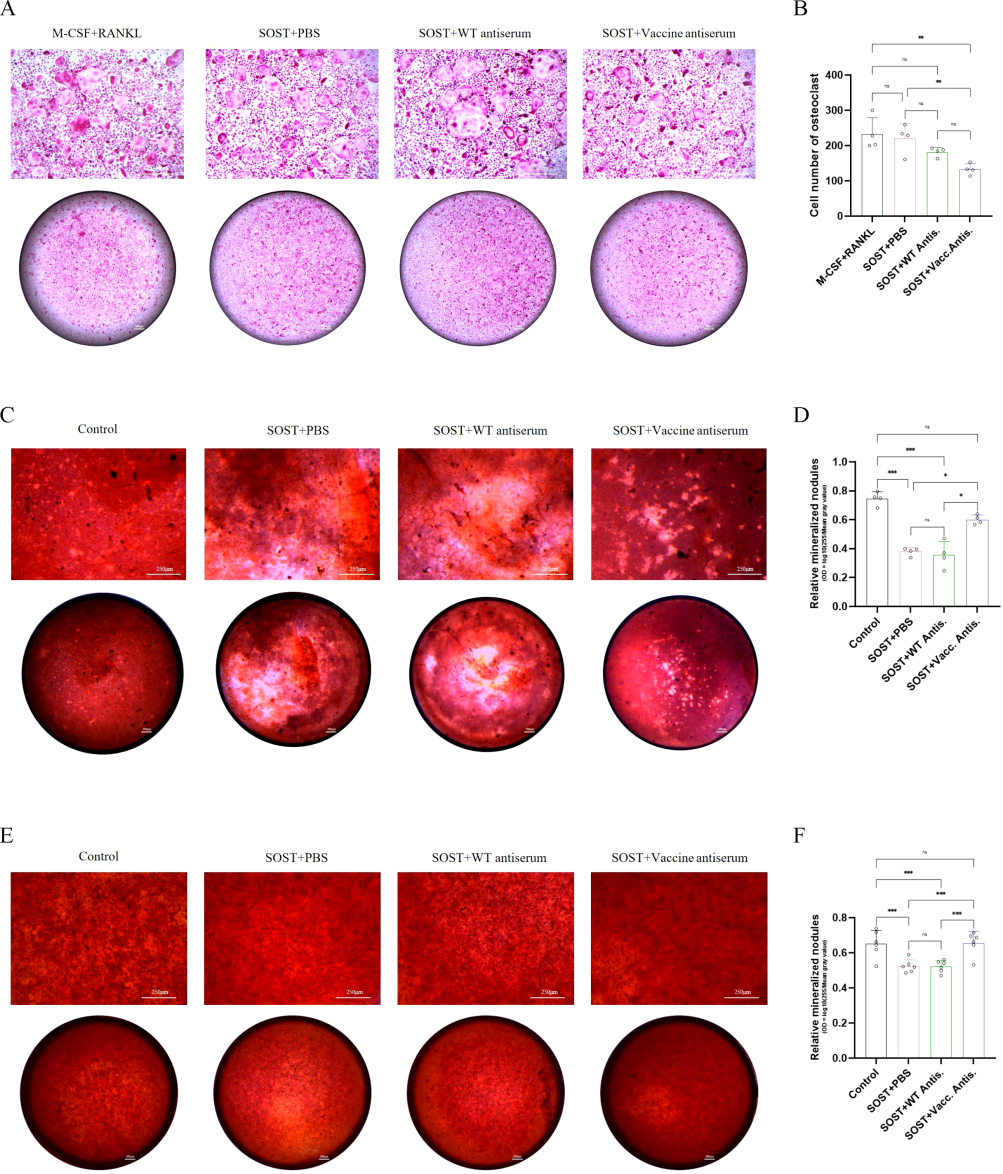
Effects of anti-SOST antiserum derived from vaccine-immunized mice on osteoclast and osteoblast differentiation. **(A)** TRAP staining of bone marrow-derived macrophages treated with SOST and anti-SOST antiserum at a 1:500 dilution, demonstrating inhibition of osteoclast differentiation (n=4). **(B)** Quantification of TRAP-positive osteoclasts in **(A)**. **(C)** Alizarin Red S staining of bone marrow mesenchymal stem cell-derived osteoblasts cultured with osteogenic medium and treated with SOST and anti-SOST antiserum at a 1:100 dilution, indicating enhanced osteoblast differentiation and mineralization upon vaccine antiserum treatment (n=4). **(D)** Quantitative analysis of mineralization in **(C)**. **(E)** Alizarin Red S staining of MC3T3-E1 subclone 14 cells cultured with osteogenic medium and treated with SOST and vaccine antiserum at a 1:100 dilution, showing rescue of SOST-mediated inhibition of osteoblast differentiation and mineralization (n=6). **(F)** Quantitative analysis of mineralization in **(E)**. Scale bars, 250 μm and 500 μm. Data are expressed as mean ± SD; ^*^p < 0.05, ^**^p < 0.01, ^***^p < 0.001; ns, not significant. Statistical significance was determined by one-way ANOVA followed by Tukey’s multiple comparisons test.

The antiserum obtained from vaccinated mice, which exhibited the highest antibody titer, was selected for *in vitro* functional assays using primary osteoclasts and osteoblasts. To model the *in vivo* role of SOST, which promotes osteoclast differentiation and inhibits osteoblast maturation, recombinant SOST was co-cultured with the respective cell types. Results showed that SOST supplementation had no sinificantly effect on osteoclast differentiation and maturation, however, the addition of the antiserum significantly attenuated osteoclast differentiation and maturation, leading to a marked reduction in osteoclastogenesis (**Figures 11 A, B**). In primary osteoblasts (**Figures 11C, D**) and the MC3T3-E1 subclone 14 cell line (**Figures 11E, F**), SOST partially suppressed differentiation and mineralization; however, the presence of antiserum mitigated these inhibitory effects, thereby restoring osteoblast mineralization capacity (**Figures 11C–F**). These findings demonstrate that the vaccine-induced antiserum effectively inhibits osteoclast activity and enhances osteoblast function, confirming its functional efficacy. Moreover, these results provide preliminary evidence supporting the vaccine’s potential as a therapeutic strategy for osteoporosis.

## 4 Discussion

Osteoporosis represents a significant global public health challenge, with osteoporotic fractures incurring substantial economic costs and imposing considerable demands on individual healthcare resources and societal medical systems (1, 2). Among the available anti-osteoporotic treatments, ROMO is noteworthy for its significant ability to increase bone mass (37). Nevertheless, its high cost and strict eligibility criteria, which restrict its use to patients with diagnosed osteoporosis, hinder its broader applicability for early prevention strategies (5, 38). The potential risk of cardiovascular adverse events associated with ROMO raises important safety concerns that warrant careful consideration (38).

Recent advancements in vaccine immunology have effectively demonstrated the potential of active immunization strategies to stimulate endogenous antibody production across various chronic conditions, including ankylosing spondylitis (39), hypertension (40), diabetes (41), Alzheimer’s disease (42), and so on. Building on these innovations, our team has focused on developing a vaccine-based immunotherapy for osteoporosis (10, 11). This approach aims to achieve sustained regulation of bone formation and resorption through proactive immunization, offering a cost-effective solution that could enhance both early prevention and adjunctive long-term treatment of advanced osteoporosis.

In this study, we present an innovative osteoporosis vaccine targeting the SOST epitope (SOST_131-163_), which was identified through ROMO screening. The SOST_131–163_ epitope, situated within the loop3 region (amino acids 134–163 in SOST (43), including a 23-amino acid signal peptide), partially overlaps with previously identified antibody-binding sites in both loop2 and loop3 (43–45). However, we did not detect specific binding sites in loop2, likely due to challenges in preserving the native three-dimensional structure during separate synthesis. While ROMO effectively inhibits SOST by targeting both loops and demonstrates substantial anti-osteoporosis benefits, this broad inhibition may elevate the risk of cardiovascular side effects (5, 6). In contrast, our targeted vaccine approach focuses solely on loop3, which promotes bone formation while preserving cardiovascular health (45–47). Thus, our vaccine strategically aims to enhance bone mass while ensuring cardiovascular safety, offering a promising option for osteoporosis management.

In vaccine design, antigenicity and immunogenicity are crucial for eliciting robust and specific immune responses. Our study reveals that the SOST_131–163_ epitope contains six CTL epitopes, five HTL epitopes, two linear B-cell epitopes, and two conformational B-cell epitopes, highlighting its considerable immunogenic potential to activate both T-cell-mediated immunity and B-cell antibody production. To overcome immune tolerance associated with autologous protein vaccines, we utilized DTT protein as a carrier to enhance immunogenicity, which effectively expanded specific helper T-cell populations and promoted the differentiation and proliferation of polysaccharide-specific B cells. The candidate vaccines, DS_1_-DS_5_, successfully disrupted immune tolerance and elicited robust antibody responses in immune simulations. Notably, the DS_3_ and DS_5_ vaccines produced unique profiles by generating IgM, IgG1, and IgG2 antibodies, while the other candidates primarily generated IgM and IgG1, lacking IgG2. Given the clinical efficacy of ROMO as an IgG2 monoclonal antibody (21), our primary goal was to stimulate endogenous IgG2 antibody production, similar to ROMO. Consequently, we selected DS_3_ and DS_5_ for further exploration of their immunological mechanisms and potential applications. In selecting the immunological scaffold, we directly employed DTT to facilitate the overcoming of immune tolerance, informed by our prior findings (10). Nonetheless, the considerable potential of alternative scaffolds warrants further investigation to enhance vaccine efficacy and optimize antibody titers.

Structural analysis revealed that 94.3% of residues in the DS_3_ vaccine and 93.7% in the DS_5_ vaccine occupied favorable regions, indicating high modeling quality. Bioinformatics assessments further demonstrated these vaccines’ strong antigenicity, favorable physicochemical properties, and non-allergenic nature, establishing them as promising vaccine candidates. Molecular docking studies showed that both DS_3_ and DS_5_ vaccines effectively bind to Toll-like receptor 2 (TLR2), thereby activating this receptor and facilitating the induction of both humoral and cellular immune responses. TLR2, expressed in dendritic cells and involved in bone metabolism through the mechanism of osteoimmunology (48), is critical for osteoporosis management, as its activation inhibits inflammatory osteoclast differentiation and mitigates bone loss (49, 50). Experimental validation confirmed the successful construction of DS_3_ and DS_5_ vaccines using recombinant plasmids, with efficient expression in E. coli. The purified DS_3_ and DS_5_ vaccines elicited a significant production of anti-SOST antibodies in immunized mice, demonstrating their efficacy in overcoming immune tolerance. Further cellular assays confirmed that sera from vaccinated mice contain anti-SOST antibodies capable of inhibiting osteoclast activity and promoting osteoblast function, thereby restoring the balance between bone resorption and formation disrupted in osteoporosis. These findings highlight the potential of these vaccines as promising immunotherapeutic strategies for the prevention and treatment of osteoporosis.

## 5 Conclusions

This study introduces a SOST-targeted vaccine specifically designed for osteoporosis, demonstrating several advantages: (1) high specificity for the loop 3 domain of SOST, which may confer protective effects against osteoporosis while minimizing cardiovascular side effects; and (2) robust immunogenicity coupled with favorable physicochemical properties, effectively inducing endogenous ROMO-like antibodies. Preliminary *in vivo* experiments confirm the vaccine’s ability to overcome immune tolerance and elicit SOST-specific antibodies in murine models. Additionally, *in vitro* analyses reveal that the generated antiserum can inhibit osteoclast differentiation and enhance osteoblast activity, underscoring its therapeutic potential for osteoporosis. This innovative strategy offers a promising approach for early prevention and sustained management of the disease. Future investigations will aim to validate vaccine’s efficacy and safety *in vivo*, facilitating its progression toward clinical application.

## Data Availability

The original contributions presented in the study are included in the article/**Supplementary Material**. Further inquiries can be directed to the corresponding author.

## Supplementary material

Supplementary Figure 1 Interaction of SOST_131–163_ fragment with ROMO light and heavy chains. **(A)** SOST_131–163_ fragment docked with ROMO light chain, forming four hydrogen bonds and 105 non-bonded contacts. **(B)** SOST_131–163_ fragment docked with ROMO heavy chain, establishing two salt bridges and 90 non-bonded contacts.

Supplementary Table 1 Structure information of DS_3_ after refinement using Galaxyrefine.

Supplementary Table 2 Structure information of DS_5_ after refinement using Galaxyrefine.

Supplementary Table 3 Prediction of cytotoxic T lymphocyte and helper T lymphocyte epitopes for DS_3_ and DS_5_ vaccine.
