# Supplementary material for: In silico design of novel precision vaccine targeting sclerostin epitopes for osteoporosis prevention and treatment
Source: Front Immunol. 2025 Dec 1;16:1644437. doi: 10.3389/fimmu.2025.1644437 (PMC12702773; doi:10.3389/fimmu.2025.1644437)
Supplement: Supplementary Figure 1 — Interaction of SOST131–163 fragment with ROMO light and heavy chains. (A) SOST131–163 fragment docked with ROMO light chain, forming four hydrogen bonds and 105 non-bonded contacts. (B) SOST131–163 fragment docked with ROMO heavy chain, establishing two salt bridges and 90 non-bonded contacts. [file Table1.docx]

Table S1 Structure information of DS_3_ after refinement using Galaxyrefine

| Model | GDT-HA | RMSD | MolProbity | Clash score | Poor rotamers | Rama favored |
| --- | --- | --- | --- | --- | --- | --- |
| Initial | 1.0000 | 0.000 | 3.711 | 37.8 | 9.1 | 58.7 |
| MODEL 1 | 0.9007 | 0.580 | 1.459 | 3.7 | 0.4 | 95.7 |
| MODEL 2 | 0.8916 | 0.595 | 1.432 | 3.7 | 0.0 | 96.0 |
| MODEL 3 | 0.8816 | 0.619 | 1.306 | 5.6 | 0.4 | 98.3 |
| MODEL 4 | 0.8965 | 0.570 | 1.505 | 4.6 | 0.4 | 96.0 |
| MODEL 5 | 0.8775 | 0.640 | 1.537 | 5.4 | 0.4 | 96.3 |

GDT-HA, global distance test high accuracy; RMSD, root-mean-square deviation; Rama favored, ramachandran favored
