# Supplementary material for: In silico design of novel precision vaccine targeting sclerostin epitopes for osteoporosis prevention and treatment
Source: Front Immunol. 2025 Dec 1;16:1644437. doi: 10.3389/fimmu.2025.1644437 (PMC12702773; doi:10.3389/fimmu.2025.1644437)
Supplement: Supplementary Table 2 — Structure information of DS5 after refinement using Galaxyrefine. [file Table2.docx]

Table S2 Structure information of DS_5_ after refinement using Galaxyrefine

| Model | GDT-HA | RMSD | MolProbity | Clash score | Poor rotamers | Rama favored |
| --- | --- | --- | --- | --- | --- | --- |
| Initial | 1.0000 | 0.000 | 3.686 | 53.3 | 6.6 | 67.7 |
| MODEL 1 | 0.9424 | 0.480 | 1.772 | 10.2 | 0.0 | 96.4 |
| MODEL 2 | 0.9404 | 0.478 | 1.761 | 9.3 | 0.0 | 96.1 |
| MODEL 3 | 0.9313 | 0.484 | 1.709 | 8.7 | 0.3 | 96.4 |
| MODEL 4 | 0.9385 | 0.466 | 1.733 | 9.9 | 0.3 | 96.6 |
| MODEL 5 | 0.9294 | 0.490 | 1.606 | 9.2 | 0.0 | 97.4 |

GDT-HA, global distance test high accuracy; RMSD, root-mean-square deviation; Rama favored, ramachandran favored
