# Supplementary figures and images for: In silico design of novel precision vaccine targeting sclerostin epitopes for osteoporosis prevention and treatment
Source: Front Immunol. 2025 Dec 1;16:1644437. doi: 10.3389/fimmu.2025.1644437 (PMC12702773; doi:10.3389/fimmu.2025.1644437)

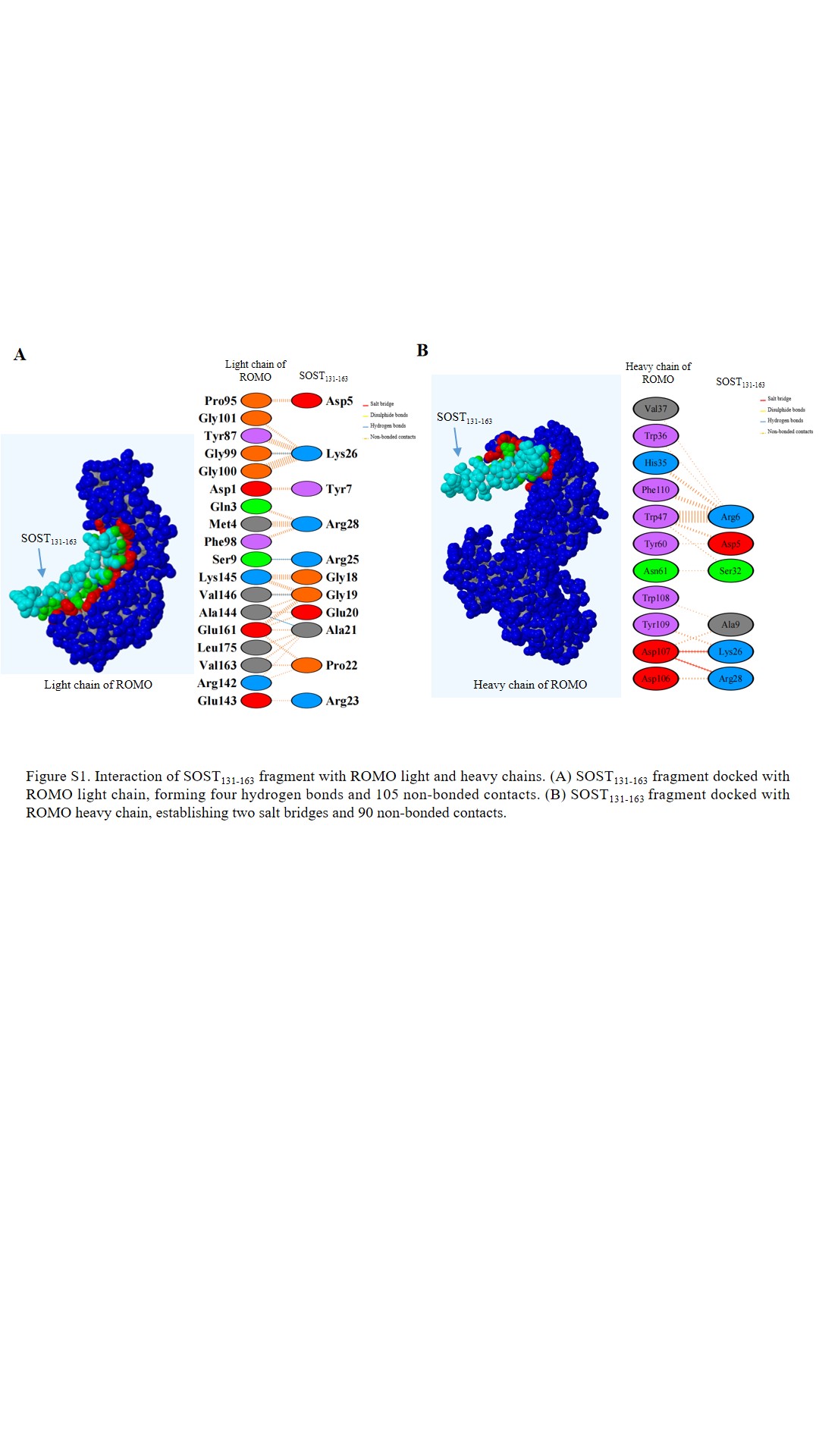

Supplement: Supplementary file 4 [file Image1.jpeg]
